# Randomized Trial of a Sexual Health Video Intervention for Black and Hispanic Adolescent Females

**DOI:** 10.1007/s11121-023-01499-0

**Published:** 2023-02-03

**Authors:** Eric Jenner, Sarah Walsh, Catherine Henley, Hilary Demby, Rebekah Leger, Gretchen Falk

**Affiliations:** The Policy & Research Group, 8434 Oak Street, 70118 New Orleans, LA USA

**Keywords:** Adolescent, Hispanic, Black, Randomized trial, Sexual health, Entertainment education, Intervention

## Abstract

Despite significant declines, adolescent birth rates in the USA are higher than other industrialized countries, with black and Hispanic youth disproportionately affected. This study assessed the efficacy of a single-session, entertainment-education sexual health video intervention for these populations. Using an individual-level randomized controlled trial, 1770 18- to 19-year-old black and Hispanic females were assigned to watch Plan A (*n* = 886) or a control video (*n* = 884) prior to a sexual reproductive health (SRH) visit. Participants self-reported data at baseline and 3 months post-baseline. Within an intent-to-treat framework, we estimated the average causal effect of assignment to Plan A on three confirmatory and five exploratory outcomes. We found that individuals assigned to Plan A had higher contraceptive knowledge, may be more likely to get sexually transmitted infection (STI) testing, and may have elevated HIV/STI risk perceptions 3 months post-video. Although we found no difference in long-acting reversible contraception (LARC) use nor frequency of condomless sex in the full sample, we did observe that first-time SRH visitors assigned to Plan A had a higher probability of using LARC than those in the control group. This study demonstrates that Plan A is a low-burden, inexpensive, and highly scalable video intervention for black and Hispanic adolescent females that has significant and borderline significant effects on protective sexual health behaviors and important antecedents. It adds to the evidence base of effective teen pregnancy prevention programs and the limited set of rigorous and causal studies investigating the effectiveness of entertainment-education interventions on sexual risk reduction. Registered in ClinicalTrials.gov (NCT03238313) on August 3, 2017.

**Supplementary Information:**

The online version contains supplementary material available at 10.1007/s11121-023-01499-0.

## Background

The adolescent birth rate in the USA remains higher than in most industrialized nations, especially in some racial and ethnic groups (Sedgh et al., 2015). In 2020, the birth rates for Hispanic and non-Hispanic black teens were 24.4 and 23.5 per 1000 females aged 15–19, respectively, more than twice that of non-Hispanic white teens (Osterman et al., 2022). Since 2009, health officials have prioritized the development of successful teen pregnancy prevention (TPP) strategies and funded an evidence review to identify effective TPP programs (Goesling et al., 2014). However, most of the TPP programs included were time- and resource-intensive (Mathematica Policy Research, 2016). Additionally, programs targeted younger teens, although 76% of teen births occur in 18- and 19-year-old females (Osterman et al., 2022). To address these gaps, the Office of Adolescent Health (OAH) (now the Office of Population Affairs) funded a round of research in 2015 to identify and study new programs.

Entertainment education (EE), which is a theory-based communication strategy that leverages the power of storytelling for health promotion and behavior change, has emerged as one promising but under-evaluated strategy (Singhal et al., 2013). A number of sexual and reproductive health (SRH) EE interventions have shown favorable effects on family planning (Rogers et al., 1999; Vaughan et al., 2000), sexual behaviors (Jones et al., 2013; Neumann et al., 2011; O’Donnell et al., 1995; Orozco-Olvera et al., 2019), human immunodeficiency virus (HIV) prevention and care (Fisher et al., 2011; Kim et al., 2019; Neumann et al., 2018), sexually transmitted infection (STI) prevention and acquisition (Downs et al., 2018; Neumann et al., 2011; Warner et al., 2008), and important antecedents to these behaviors (Wang & Singhal, 2016). Safe in the City, VOICES/VOCES, and Taking Care of Me are all single-session video interventions with some evidence of the potential effectiveness of the videos on targeted sexual health behaviors and outcomes (Harshbarger et al., 2012; Neumann et al., 2011, 2018; O’Donnell et al., 1995; Warner et al., 2008). However, the evidence base of SRH EE interventions is limited by the quality and rigor of the research conducted (Orozco-Olvera et al., 2019). Only a few evaluations have assessed low-burden SRH EE interventions using a randomized controlled trial (RCT) (Downs et al., 2018; Jones et al., 2013; Kim et al., 2019; O’Donnell et al., 1995; Warner et al., 2008).

### Purpose

This article presents findings from the RCT that assessed the efficacy of a low-burden, scalable 23-min SRH EE video intervention known as Plan A (www.myplana.org). The video was developed in 2016 with OAH funding to promote effective contraceptive use, the use of dual methods of protection, and HIV/STI testing. Through a series of three inter-related, soap opera-style vignettes, five primary topics are addressed in the video: (1) pregnancy, STI, and HIV risk perception; (2) contraceptive options, with an emphasis on long-acting reversible contraception (LARC); (3) condom use and partner negotiation skills; (4) importance of regular HIV/STI testing; and (5) comfort discussing sexual history, HIV/STI testing, and contraceptive methods with a health provider. Between each vignette, there is a short, animated sequence to deliver information about intrauterine devices (IUDs) and the implant (Plant et al., 2019). All content included in the video went through a medical accuracy review process to confirm the information presented was factually accurate and up-to-date.

Plan A was developed specifically for Hispanic and black female adolescents to promote equitable access to SRH information and reduce disparities in adolescent sexual health risk and birth rates across these racial and ethnic groups. The main characters featured in the film all identify as either black and/or Hispanic. The video is intended to be shown immediately before an appointment with a SRH provider and aims to motivate behavior change. It provides facts about contraceptive and sexual protection options to create awareness, demonstrates risk reduction strategies for unwanted pregnancy and HIV/STIs, and models effective communication with sexual partners and SRH providers. Plan A was built upon previous health EE videos, which have some evidence of motivating positive behavior change (Neumann et al., 2018) and reducing incident STIs (Harshbarger et al., 2012; Warner et al., 2008). Plan A is grounded in the extended elaboration likelihood model (E-ELM) and social cognitive theory (SCT) (Bandura, 2004; Slater & Rouner, 2002).

SCT posits that people learn, can be motivated to change, and develop a belief about their ability to change, by observing others (Bandura, 2004). The developers of the Plan A video hypothesize that viewers learn by watching characters make contraceptive and sexual protection choices and experience the consequences of those decisions vicariously. In this way, viewers’ outcome expectations may be influenced and result in behavior change. The E-ELM is a dual-process model that explains how different types of persuasive messages may be processed, depending on the nature of the message (Petty & Cacioppo, 1986; Slater & Rouner, 2002). As it relates to entertainment education, the model postulates that viewers who are engaged and entertained will be less inclined to counter-argue with persuasive messages. The developers expect that Plan A’s pro-health messages and values will appeal to viewers in part because of the video’s high production value. They also expect viewers to process these messages peripherally and favorably because they are subsumed within a dramatic and engaging storyline, and conveyed by relatable, sympathetic, and identifiable characters. According to E-ELM, character identification is a key facilitator of increased absorption and acceptance.

Confirmatory research questions of the RCT asked whether the offer to view Plan A differentially impacted treatment participants’ use of LARC, frequency of condomless sex, and receipt of STI testing 3 months after being assigned to watch the video as compared to control participants and were prespecified in a statistical analysis plan registered on clinicaltrials.gov. Exploratory research questions asked whether the offer to watch Plan A impacted participants’ contraceptive knowledge, pregnancy and HIV/STI risk perception, and HIV/STI testing. Each of these outcomes was investigated because they are central to the intervention’s theory of change and are salient in the video content. The video incorporates medically accurate contraceptive information as part of the dialogue in the narrative (knowledge). Plan A also emphasizes the importance of both routine STI and HIV testing and dramatizes the risks associated with unprotected sex (risk perceptions and testing behavior). We also investigated the impact of Plan A on LARC use for first-time SRH users because the developer of Plan A was particularly interested in this outcome for this group, and evidence suggests that contraceptive use behaviors are persistent and may be more resistant to change than other SRH decisions (Demaria et al., 2019; Fekadu & Kraft, 2001). First-time SRH clinic visitors may be more responsive to persuasive change messages (i.e., LARC uptake) because their behaviors are not yet habituated. Finally, to better understand the immediate effect of the video on the SRH visit occurring directly after viewing, we evaluated data provided by participants about the content of their discussion with the provider and their comfort and satisfaction with the visit.

## Methods

### Study Design and Setting

The study, an individual-level RCT, was conducted between June 2016 and June 2020 in eight Planned Parenthood (PP) health centers across California: five in California’s San Joaquin Valley and three in the greater Oakland area. These areas experience some of the highest teen birth rates in California (California Department of Public Health, 2015). Health centers were selected that served large populations of Hispanic and/or black adolescents.

### Recruitment, Eligibility Screening, and Enrollment

To be eligible for enrollment, individuals had to meet the following inclusion criteria: self-identify as female; be 18 or 19 years old; self-identify as Hispanic and/or black; be visiting a study health center; be deemed appropriate for the study by PP staff with regard to physical and mental health; indicate that they were not knowingly pregnant nor trying to become pregnant; and not previously enrolled in this or another TPP study. Study coordinators (PP staff) reviewed patient demographic information for upcoming appointments at study health centers and screened all 18- and 19-year-old female patients for eligibility. Only individuals who met the study eligibility criteria and provided informed consent were enrolled. Enrollment continued until the required sample size was reached based on pre-implementation power calculations.

### Randomization and Contrast

Eligible participants were randomly assigned to each condition at a 1:1 ratio prior to receiving the baseline questionnaire. Random assignment was stratified by four administrative regions in blocks of varying sizes using the *ralloc* command in Stata 14.2. A senior research analyst created and maintained the master randomization list. Participants assigned to treatment were given the opportunity to watch Plan A. Participants assigned to control were given the opportunity to watch a 17-min video about the hazards of cigarettes, which included no SRH content. Questionnaires were administered electronically using SNAP surveys, and videos were screened on Chromebooks that were provided to participants by study coordinators. When the participant clicked the “Submit” button after completing the baseline questionnaire, Plan A or the control video began based on the participant’s randomly assigned condition. Both videos were screened through the Wistia platform, which collected individual-level video dosage data. One of the treatment participants received the control video by mistake, and one of the control participants was not given the opportunity to watch the control video; given that our benchmark approach pre-specified an intent-to-treat (ITT) analysis, these two individuals were retained in the final impact analysis within their originally assigned condition.

### Data Collection

After randomization, study coordinators administered the baseline questionnaire to participants. The instrument contained 75 items that measured contraceptive knowledge and asked participants to self-report background characteristics, contraceptive use, sexual behaviors and experiences, the belief of risk, and intentions related to sexual behaviors.

After having the opportunity to watch their assigned video, participants went to their scheduled SRH appointment. Immediately after the appointment, they were asked to complete a post-visit questionnaire, which contained seven closed-ended items that asked about topics, behaviors, and questions discussed with the provider during the visit, how comfortable they felt talking about these issues with their provider, and their general satisfaction with the visit.

Three months post-enrollment, research assistants contacted participants to administer follow-up questionnaires. The follow-up questionnaire was almost identical to the baseline questionnaire; it included all 75 questions plus two additional items that asked how and where the participant completed the questionnaire. Follow-up windows opened 3 months after the baseline enrollment date and, once the window opened, participants had 4 months to complete each questionnaire.^1^ Most participants (86.6%) completed the follow-up questionnaire during the first month of the window. Participants were given a gift card incentive when they completed a questionnaire and responded to requests to update their contact information. All data collection procedures were the same at each follow-up time point and identical for both treatment and control participants. Non-participation or failure to obtain follow-up data was primarily the result of study staff not being able to reach participants after they enrolled in the program.

### Confirmatory and Exploratory Measures

Three confirmatory behavioral outcomes were assessed: LARC use, sex without a condom, and STI testing. Operational definitions for these confirmatory outcomes, all inclusion criteria, and the analytic framework and procedures to estimate the efficacy of plan A were prespecified prior to outcome data collection. LARC use was operationalized as a dichotomous variable; participants were coded as either (1) not currently using or (2) using an intrauterine device (IUD) or implant. Times having sex with no condom in the past 3 months was constructed as a count variable that quantified the number of times a participant reported not using condoms while engaging in any type of sex (vaginal, oral, or anal) over the past 3 months. Participants who reported always using condoms during each sex act or no sex in the past 3 months were coded as zero. STI testing in the past 3 months was constructed as a dichotomous variable where participants were coded as either having been tested for STIs in the past 3 months or not.

Exploratory measures included contraceptive knowledge, HIV/STI risk perception, pregnancy risk perception, HIV testing, and post-visit questionnaire items. Contraceptive knowledge was measured with 10 true or false questions; scores represent the proportion of items to which the participant provided an accurate response. Pregnancy risk perception and HIV/STI risk perception were each assessed with two items using a seven-point semantic-differential scale that asked participants to indicate how likely they thought certain events would happen over the course of a year if they engaged in specific risk behaviors; a low score indicates low perceived likelihood and a high score indicates high likelihood. HIV testing in the past 3 months was constructed identically to the STI testing measure.

Post-visit questionnaire items that asked about topics discussed with the provider during the health visit were used to construct nine binary variables with participants coded as discussed or not discussed. The final three items pertaining to comfort level and satisfaction with the provider were each assessed with one item using a five-point semantic-differential scale; a low score indicates low comfort/satisfaction and a high score indicates high comfort/satisfaction.

### Data Management

Data screening identified invalid, unreliable, outlying, and inconsistent values.^2^ For each outcome sample, we employed case-wise deletion for any respondent who failed to provide complete responses to all items necessary for the outcome measure; no outcome data were imputed. Baseline equivalence statistics on unimputed background characteristics for each of the three confirmatory outcome measure samples are provided in Table 1. For our impact analysis, missing baseline covariate data were imputed using dummy variable adjustment, where continuous variables missing a value were imputed to the mean for the sample, and dichotomous variables missing a value were imputed to zero. We created indicators to identify records where imputation had been used to address missing baseline values.

**Table 1:** Baseline characteristics and equivalence of treatment and control groups, by confirmatory outcome samples

	**LARC use *N* = 1700**^**a, b**^			**Times having sex without condoms *N* = 1694**^**a, c**^			**STI test *N* = 1697**^**a, d**^		
**Characteristic**	**Treatment *n* = 855**	**Control *n* = 845**	**Standardized difference**	**Treatment *n* = 852**	**Control *n* = 842**	**Standardized Difference**	**Treatment *n* = 853**	**Control *n* = 844**	**Standardized difference**
Mean age (years)	19.04	19.03	0.003	19.04	19.03	0.002	19.04	19.03	0.007
Black/African American^e^	15%	13%	0.064	15%	14%	0.063	15%	14%	0.065
Hispanic/Latina^e^	87%	88%	–0.051	87%	88%	–0.051	87%	88%	–0.052
High school education	94%	93%	0.114	94%	93%	0.104	94%	93%	0.114
Sexual initiation	98%	99%	–0.219	98%	99%	–0.218	98%	99%	–0.220
Current LARC use	9%	10%	–0.079	-	-	-	-	-	-
Mean times having sex without condoms in the past 3 months	-	-	-	17.5	19.0	–0.044	-	-	-
STI test in the past 3 months	-	-	-	*-*	*-*	-	26%	24%	0.057

^a^Sample sizes reported reflect the number of participants in the analytic sample for each outcome (i.e., the number of participants who have a value for the confirmatory outcome measure at the 3-month follow-up). However, the sample sizes do not necessarily indicate the number of participants who provided a response to the baseline characteristic being assessed in this table. For most characteristics, the analytic sample size is equivalent to the sample size reporting a value for the baseline characteristic, but slight variations do exist due to missingness. Statistics in this table reflect non-imputed baseline characteristic values

^b^Of the 884 participants randomized to the control condition and 886 participants randomized to the treatment condition, 39 control participants and 31 treatment participants did not provide data at the 3-month follow-up time point for the construction of the LARC use measure

^c^Of the 884 participants randomized to the control condition and 886 participants randomized to the treatment condition, 42 control participants and 34 treatment participants did not provide data at the 3-month follow-up time point for the construction of the times having sex without condoms measure

^d^Of the 884 participants randomized to the control condition and 886 participants randomized to the treatment condition, 40 control participants and 33 treatment participants did not provide data at the 3-month follow-up time point for the construction of the STI test measure

^e^Race and ethnicity were assessed at eligibility screening with the question “What is your race and ethnicity?” Available responses included: White; Black or African American; Hispanic, Latino or Spanish origin; American Indian or Alaska Native; Asian; Native Hawaiian/other Pacific Islander; Unknown; Some other race/ethnicity (specify). Individuals were allowed to select as many responses as applied to them

### Analysis

Within an ITT framework, we used an available case analysis to identify the average causal effect of being assigned to watch Plan A. Equivalence was assessed using the standardized mean difference of treatment and control groups at baseline for a set of predetermined variables (age, race, ethnicity, education, sexual initiation, and the baseline measure of the outcome). For analytic and interpretive simplicity, we initially prespecified the use of ordinary least squares regression to estimate the average treatment effect for all three confirmatory outcomes (see Analysis Plan registered on clinicaltrials.gov: NCT03238313). However, because some results are sensitive to model choice, we have elected to present results in terms of the negative binomial and logistic regression models that more closely meet the distributional characteristics of the data (Hilbe, 2014). Finally, we converted the treatment coefficients to marginal effects calculated at the mean value for all covariates.

Although a difference of means should produce an unbiased estimate of the effect of Plan A on outcomes, we included the following covariates in the regression equations to improve the efficiency of the estimates: age, black race, Hispanic ethnicity, high school education, and the baseline measure of the outcome variable being estimated. We also included imputation indicators and regional dummy variables to account for blocking. All covariates were mean-centered. All analyses were conducted in Stata 16.1. We conducted sensitivity analyses (Supplementary Material), otherwise employing the benchmark approach, to test whether findings are sensitive to data management and analytic decisions.

## Results

**Fig. 1:**
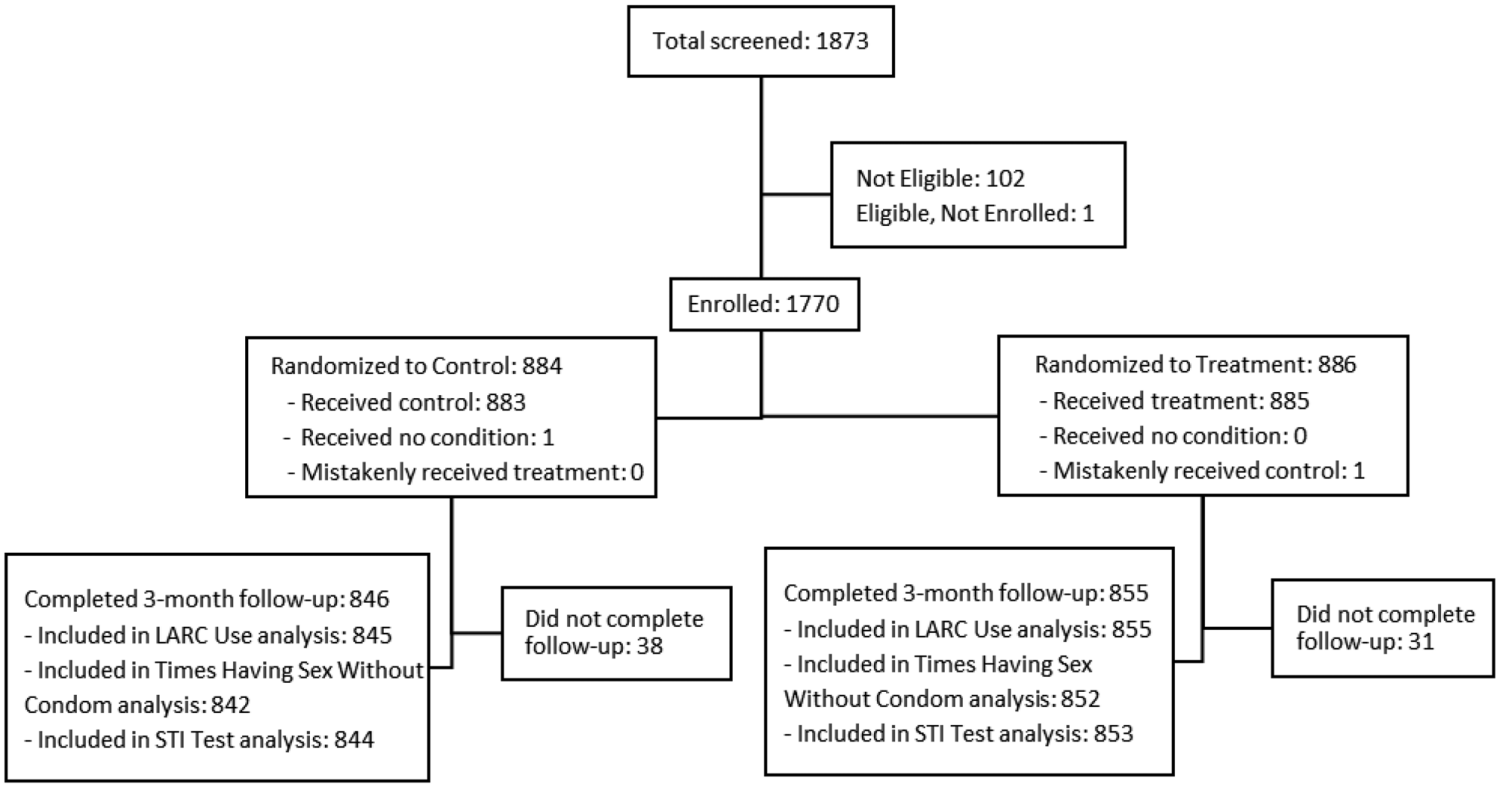
Flow diagram of screening, enrollment, randomization, and follow-up

Between June 2016 and June 2019, 1770 females were enrolled, with 884 participants randomized to control and 886 assigned to Plan A. The analytic samples for the confirmatory outcome measures vary slightly due to item non-response, with 1700 females in the assessment of LARC use, 1694 females in the assessment of times having sex without condoms, and 1697 females in the assessment of STI testing. The overall attrition for all three confirmatory outcomes is 4%, and the differential attrition is < 1% (Fig. 1).

Baseline characteristics are similar in all samples. Participants were, on average, 19 years old. The majority identified as Hispanic (87%); 14% identified as black. Most had already completed high school (93%) and reported sexual initiation (98%). Approximately 10% of participants were currently using LARC at enrollment, 25% had been tested for STIs in the past 3 months, and were having sex without condoms, on average, 18 times in the past 3 months. Baseline equivalence statistics indicate that treatment and control groups are well balanced on observed characteristics (Table 1).

Findings demonstrate that Plan A has no statistically detectable effect on LARC or condom use for the full sample (Table 2). However, Plan A may be having a meaningful effect on STI testing. Statistical uncertainty remains—the unadjusted treatment effect is significant (*p* value = 0.038) and the pre-specified covariate-adjusted model is borderline (*p* value = 0.053). Marginal effects generated from the covariate-adjusted model suggest that the treatment group has a 4.8% greater predicted probability of getting STI tested in the past 3 months than the control group. Three months after the intervention, Plan A participants also have borderline elevated perceptions of HIV/STI risk (*p* value = 0.057) and may also have a greater probability of getting HIV tested (*p* value = 0.073).

**Table 2:** Findings for confirmatory and exploratory outcomes, by benchmark approach and unadjusted model, three months post-baseline

	Model 1: Benchmark approach			Model 2: Unadjusted		
Variable	Effect estimate (standard error)^a^	*p* value	Marginal effect^b^	Effect estimate (standard error)^a^	*p* value	Marginal effect^b^
Behavioral outcomes						
Current LARC use^c^	0.17 (0.145)	0.230	0.022	0.08 (0.128)	0.512	0.012
Times having sex without condoms in past 3 months^c^	–0.04 (0.063)	0.504	–0.663	–0.08 (0.076)	0.296	–1.541
STI test in the past 3 months^c^	0.19 (0.099)	0.053	0.048	0.20 (0.097)	0.038	0.050
HIV Test in the past 3 months	0.19 (0.105)	0.073	0.043	0.18 (0.101)	0.077	0.041
Current LARC use (first-time SRH users only)	0.53 (0.259)	0.040	0.069	0.56 (0.256)	0.029	0.074
Behavioral antecedents						
Contraceptive knowledge	0.03 (0.007)	< 0.001	0.029	0.04 (0.009)	< 0.001	0.038
HIV/STI risk perception	0.18 (0.093)	0.057	0.177	0.25 (0.110)	0.026	0.246
Pregnancy risk perception	0.07 (0.079)	0.389	0.068	0.10 (0.086)	0.227	0.104

^a^Effect estimates listed are the coefficients produced by the analytic models. Logistic regression models produced the coefficients for current LARC use, STI test in the past 3 months, and HIV test in the past 3 months, a negative binomial model produced the coefficient for times having sex without condoms in the past 3 months, and ordinary least squares regression models produced the coefficients for contraceptive knowledge, HIV/STI risk perception, and pregnancy risk perception

^b^Marginal effects are calculated to provide the predicted probability of the outcome of interest at the mean value for all covariates included in each model

^c^Denotes confirmatory outcomes

Effect estimates further indicate that Plan A is having a small but significant effect on contraceptive knowledge among treatment participants (*p* value < 0.001) and a significant effect on LARC use for first-time SRH users (*p* value = 0.040). Marginal effects show that for the typical first-time SRH user who was offered Plan A, the estimated probability of using LARC 3 months post-video was 6.9% greater than for a control participant.

Analysis of post-visit questionnaire data suggest that participants who were offered Plan A discussed IUDs, implants, condom use, dual methods of protection, and sexual behavior risks with their healthcare providers at higher rates than participants offered the control condition. Plan A participants also reported experiencing greater discomfort in talking with their healthcare providers about their sexual health and behaviors and reported greater dissatisfaction with their visit (Table 3).

**Table 3:** Effect estimates for post-visit questionnaire items

Questionnaire item	*N*	Effect estimate^a^	Standard error	Marginal effect
Discussed IUDs	1621	1.20**	0.119	0.231
Discussed implants	1621	0.60**	0.101	0.149
Discussed condoms	1621	0.36**	0.101	0.088
Discussed other birth control	1621	0.08	0.102	0.020
Discussed dual methods of protection	1621	0.45**	0.104	0.105
Discussed HIV/STI testing	1621	0.18 *	0.101	0.043
Discussed sexual behavior risks	1621	0.29**	0.104	0.067
Discussed your sexual behaviors	1556	0.00	0.121	0.001
Asked questions/mentioned concerns	1560	0.02	0.105	0.005
Comfort level talking with provider about your sexual health	1611	–0.06**	0.028	–0.061
Comfort level talking with provider about your sexual behaviors	1263	–0.09**	0.036	–0.087
Satisfaction level with provider	1210	–0.06**	0.029	–0.064

*indicates *P* value < 0.10; ** indicates *P* value < 0.05

## Discussion

Causal estimates produced from this RCT provide evidence that Plan A is a promising, low-burden, brief SRH EE video intervention for black and Hispanic female adolescents who are about to receive SRH care, which may have favorable effects on protective sexual health behaviors and theoretically important antecedents in these at-risk and underserved populations. The strengths of this study are its methodological rigor—a randomized design, with a high follow-up rate (96%), low differential attrition (< 1%), and benchmark inferences that are stable across a battery of sensitivity tests and racial/ethnic subgroups. Baseline equivalence analysis further attests that randomization appears to be well implemented.

Results demonstrate that watching Plan A prior to a SRH health visit leads to increased STI testing for black and Hispanic female adolescents. Convention suggests that with a test statistic of 1.91 (*p* value = 0.053), the null hypothesis should be interpreted as true and the inference should be that the intervention is having zero effect on the outcome. This is an overzealous interpretation of what hypothesis tests can do in general (Wasserstein & Lazar, 2016), and in this case, we believe it to be an oversimplification of what the data are telling us. Statistical tests for this outcome are substantively sensitive to model specifications (Table 2; Supplementary Material—Table S2), and closely related outcomes (HIV testing) and their antecedents (STI/HIV risk perception) all demonstrate patterns that are consistent with meaningful STI testing increases. Taken together and consistent with the intervention’s theory of change, the evidence suggests that people who view Plan A before a health visit are more likely to experience elevated perceptions of their risk for STIs and HIV and this in turn motivates them to get tested. While we cannot rule out that the effect is zero (given statistical uncertainty), this is the first and only experiment of the efficacy of Plan A and the 4.8% increase in the probability of getting tested is our best estimate of the average treatment effect (Gerber & Green, 2012). If this is accurate, the effect is modest (Cox index = .117), but when we consider the negligible burden of Plan A, the effect is noteworthy because it comes at virtually no cost to staff or the clinic. Further research, perhaps with an even larger or more targeted sample, is warranted.

Findings also indicate that Plan A appears to be imparting important SRH information, leading to a significant increase in viewers’ contraceptive knowledge 3 months after watching the video. Although no significant effects were observed in LARC use and condom use consistency within the full sample, a subgroup analysis indicates that Plan A was effective in motivating LARC uptake among black and Hispanic females receiving SRH care for the first time (*n* = 480). This aligns with literature that contends that prior risk behaviors can constrain future SRH behavior (Demaria et al., 2019; Fekadu & Kraft, 2001). If these theories are accurate, then the video may be especially persuasive for new initiates of SRH care because they may be more likely to be open to its influential message. An array of empirical work in communication and psychology finds that those who are uninitiated, uninformed, or disengaged with an object (in this case, LARC) will tend not to have formed coherent, strong, or even accessible beliefs about that object (Azjen & Sexton, 1999; Crocker et al., 1984; Hastie & Dawes, 2009; Higgins, 1996). Entertaining scenarios provided by EE may simply provide sufficient motivation to begin attending to, processing the persuasive messages, and forming supportive attitudes, beliefs, and opinions. Another line of thinking suggests that new initiates may have avoided SRH not because it was unimportant to them, but because of fear elicited by misinformation (Payne et al., 2016; Potter et al., 2014). In any case, data suggest that SRH visits are an opportune time for an EE intervention to reduce the risk for first-time clients. For this group, one 23-minute video was enough to significantly improve contraceptive knowledge and increase LARC uptake 3 months later. The question of how and why it does so is empirical and worth further investigation.

Analysis of feedback collected immediately after the SRH visit provides evidence that Plan A may also be motivating the sort of interactions hypothesized by the intervention’s theory of change, though with some predictable but perhaps unanticipated consequences. Participants assigned to Plan A report that they discuss important sexual health topics with their providers at higher rates than participants in the control group. At the same time, these participants are also reporting greater (though modest) discomfort with these discussions (Hedge’s g for comfort talking with a provider about sexual health =  −0.109; Hedge’s g for comfort talking with a provider about sexual behaviors =  −0.136). One interpretation of these findings is that participants assigned to the Plan A video are having relatively more SRH discussions with their healthcare providers and, because these discussions may be uncomfortable for some, these respondents are reporting relatively more (but small in magnitude) discomfort on average. The video, in other words, maybe motivating patients to have discussions with their providers, but it does little to help them feel more comfortable about these discussions. This is, of course, merely conjecture at this point, as the data we have collected do not permit an empirical answer to why Plan A participants report greater discomfort than those who did not see the video. Mixed methods may be best suited to explore participant and healthcare provider perspectives on the interactions that follow (viewing of) the video.

This study is limited in its scope. We have examined the efficacy of Plan A for specific populations (black and Hispanic female adolescents) within a limited geographic location (mid-California) for a particular period of time (3 months post-baseline). This sort of specification is necessary when one aims to rigorously study program impacts with finite resources. Future research could broaden this scope where it makes sense; in particular, we recommend that the long-term efficacy of the intervention be evaluated, with a specific investigation of participants’ continued engagement in SRH care after watching the video. Generalizability may also be limited by the setting of the intervention. Both Plan A and control participants received the same high standard of care (SOC) at Planned Parenthood. During the study period, participants attended one or more provider visits where the SOC included discussing their sexual health and covered a range of effective contraceptive methods. Future studies could investigate whether Plan A has a stronger impact in environments where young women do not receive the same intensity and/or quality of sexual health information, such as general health clinics, community organizations, or schools. Another limitation of the study is its reliance on self-reported data, which may not necessarily reflect actual behaviors and can lack precision and accuracy.

## Implications for Practice and Research

From a programming standpoint, these effects observed at 3 months are striking because they are the result of a brief and effortless (from the point of view of clinic staff and participants) intervention. Plan A is a brief, low-resource, and low-burden intervention for black and Hispanic female adolescents that can be easily scaled and incorporated into clinic settings; this is in contrast with many of the existing TPP interventions that are both time and resource-intensive, requiring multiple sessions and trained facilitators (Centers for Disease Control & Prevention, 2021; Mathematica Policy Research, 2016). Furthermore, the observed impact of the intervention, though modest, may be meaningful. A recently conducted systematic review of interventions to improve the uptake of STI testing in clinic-based settings indicates that strategies which result in at least a 5% increase in testing, as this study observed, are seen as moderately effective, particularly if they are low-cost (Taylor et al., 2016).

This study can contribute to the corpus of research that has investigated the effects of EE interventions on STI screening and acquisition (Downs et al., 2018; Neumann et al., 2011; Warner et al., 2008). A contemporary meta-analysis noted the paucity of high-quality, rigorous designs as a major limitation in determining the efficacy of youth-focused SRH EE interventions (Orozco-Olvera et al., 2019). This study provides causal evidence from a rigorously conducted RCT that a one-time video EE intervention can result in positive and consequential SRH-related behavior change.

### Supplementary Information

Below is the link to the electronic supplementary material.

Supplementary file1 (DOCX 86 KB)

## Data Availability

Details on how to access a deidentified, individual-level dataset from this study can be found on ClinicalTrials.gov (NCT03238313).

## Footnotes

1 Participants were given 4 months to complete follow-up questionnaires to provide sufficient time to collect data, particularly because this is a hard-to-reach population and it was anticipated that it would be difficult to collect follow-up data.

2 Our approach was to include outlying (included *n* = 137 in times having sex with no condoms analysis) and inconsistent values (included *n* = 62 in current LARC use analysis), recode invalid values to missing (none identified), and replace missing data determined at a certain time point to be unreliable (excluded *n* = 1 in all three confirmatory outcome analyses).
