# Supplementary material for: Randomized Trial of a Sexual Health Video Intervention for Black and Hispanic Adolescent Females
Source: Prev Sci. 2023 Feb 3;24(Suppl 2):262–71. doi: 10.1007/s11121-023-01499-0 (PMC10764370; doi:10.1007/s11121-023-01499-0)
Supplement: Supplementary file 1 — Supplementary file1 (DOCX 86 KB) [file 11121_2023_1499_MOESM1_ESM.docx]

**Randomized Trial of a Sexual Health Video Intervention for Black and Hispanic Adolescent Females**

**Journal:** Prevention Science

**Authors:** Eric Jenner, PhD^1^; Sarah Walsh, PhD^1^; Catherine Henley, PhD^1^; Hilary Demby, MPH^1^; Rebekah Leger, MPH^1^; Gretchen Falk, MPH^1^

**Affiliations:**

^1^ The Policy & Research Group, New Orleans, Louisiana, USA

**Corresponding Author Contact:** Catherine Henley, catie@policyandresearch.com

Supplementary Material: Video Health Study Detailed Analytic Methods

Research Questions

Confirmatory Research Questions

We investigated the following confirmatory research questions with three-month follow-up *Participant Questionnaire* data using the benchmark analytic approach described below.

1. What is the impact of the offer to watch *Plan A* (treatment) relative to the offer to watch *The Toxic Life Cycle of a Cigarette* (control) on participants’ reported use of long-acting reversible contraception (LARC) three months after receiving the treatment?
2. What is the impact of the offer to watch *Plan A* (treatment) relative to the offer to watch *The Toxic Life Cycle of a Cigarette* (control) on participants’ reported times having sex without condoms three months after receiving the treatment?
3. What is the impact of the offer to watch *Plan A* (treatment) relative to the offer to watch *The Toxic Life Cycle of a Cigarette* (control) on participants’ reported receipt of sexually transmitted infection (STI) testing three months after receiving the treatment?

A detailed description of how these confirmatory measures were constructed is provided in Table S1.

Exploratory Research Questions

In addition to the confirmatory research questions listed above, we investigated the following exploratory research questions using the same benchmark analytic approach.^[[1]](#footnote-2)^ The first five questions were explored with three-month follow-up *Participant Questionnaire* data; the sixth question was explored using *Post-visit Questionnaire* data collected from participants immediately after the baseline sexual/reproductive health (SRH) ended.

1. What is the impact of the offer to watch *Plan A* (treatment) relative to the offer to watch *The Toxic Life Cycle of a Cigarette* (control) on participants’ reported receipt of HIV testing three months after receiving the treatment?
2. What is the impact of the offer to watch *Plan A* (treatment) relative to the offer to watch *The Toxic Life Cycle of a Cigarette* (control) on participants’ reported knowledge and awareness of contraceptive options three months after receiving the treatment?
3. What is the impact of the offer to watch *Plan A* (treatment) relative to the offer to watch *The Toxic Life Cycle of a Cigarette* (control) on participants’ reported perception of their risk for pregnancy three months after receiving the treatment?
4. What is the impact of the offer to watch *Plan A* (treatment) relative to the offer to watch *The Toxic Life Cycle of a Cigarette* (control) on participants’ reported perception of their risk for HIV/STIs three months after receiving the treatment?
5. Among participants who reported attending a SRH visit for the first time at baseline, what is the impact of the offer to watch *Plan A* (treatment) relative to the offer to watch *The Toxic Life Cycle of a Cigarette* (control) on these participants’ reported use of LARC three months after receiving the treatment?
6. What are the immediate impacts of the offer to watch *Plan A* (treatment) relative to the offer to watch *The Toxic Life Cycle of a Cigarette* (control) on participants’ reported discussion topics with their provider, and their comfort and satisfaction with the SRH visit, immediately after attending the baseline SRH visit?

A detailed description of how these exploratory measures were constructed is provided in Table S1.

Data Management

Data Screening

Prior to analysis, staff systematically screened the analytic variables (baseline and outcome) to identify invalid, outlying, inconsistent, and unreliable data. The steps taken in this data cleaning process are outlined below.

Identify and Flag Invalid Responses

The first step in the data screening process was inspecting the data for instances in which responses were invalid because they were outside of a predetermined range of plausible or acceptable values.^[[2]](#footnote-3)^ Each questionnaire type (e.g., baseline, three-month follow-up) had a codebook, which was prepared by an analyst, that contained variable names, valid variable values or ranges of values, and, when applicable, value labels.^[[3]](#footnote-4)^ Referring to the codebook, a lead research analyst performed diagnostics in Stata to ensure that responses to all analytic measures were valid (i.e., data were within ranges specified in the codebook). A data analyst inspected the data using two commands in Stata. First, the analyst used the command *sum variable_name*, which provided summary statistics (mean, minimum, maximum, standard deviation) for all numeric variables. The analyst checked that the minimum and maximum values were valid. When this command revealed values out of range, the analyst then inspected the data using the command *tab variable_name, missing*, which provided a frequency table of all values (including missing values) so the analyst could identify and flag all values that were out of range. The lead analyst then followed up with study staff to determine whether the value was truly invalid (and subsequently re-coded those values to item missing) or whether the value was the result of a data entry error and could be updated to the correct value.

Identify and Flag Outliers

The second step in data screening was identifying and flagging severe outliers. Outliers (operationally defined below) are values that are extreme compared to other observations but are not plainly invalid. In this process, we inspected outliers so that we could try to ascertain whether they were in fact true (or plausible) values or potentially a result of measurement error. Our approach to identifying and flagging outliers was as follows:^[[4]](#footnote-5)^

- First, we defined values as severe outliers according to their relation to the interquartile range (IQR). Severe outliers were defined as values outside of the *outer fences* of the population distribution.
  - IQR = Q3(3^rd^ quartile or 75^th^ percentile) – Q1 (1^st^ quartile or 25^th^ percentile)
  - Upper outer fence: Q3 + 3*IQR
  - Lower outer fence: Q1 – 3*IQR
- Second, we created an outlier indicator variable, where observations deemed severe outliers were coded as 1 and all others were coded as 0.

The only variables for which we inspected outliers are those used in the construction of *Times having sex without condoms* outcome measure (times having vaginal sex without a condom, times having oral sex without a condom, times having anal sex without a condom) because they have no upper limit (all other variables used in analysis are either categorical or have predicated upper and lower bounds). We identified 77 outliers at baseline and 76 outliers in the 3-month questionnaire for times having vaginal sex without condoms. We also identified 89 outliers at baseline and 92 outliers in the 3-month questionnaire for times having oral sex without condoms. No outliers were identified in either questionnaire administration for times having anal sex without condoms.

Our benchmark analytic approach was to include data flagged as outliers in analysis, because we do not know for certain whether the values were true or invalid. However, we also ran sensitivity analyses that excluded these data from the construction of applicable outcome measures (see Table S2 for results).

Identify and Flag Inconsistencies in Reporting of Sexual Behaviors

The third step in data screening was inspecting the data and identifying internal inconsistencies in sexual behavior outcome data.^[[5]](#footnote-6)^ Internal inconsistencies refer to discrepancies in responses (to related questions) in the same questionnaire administration.^[[6]](#footnote-7)^ In order to minimize internal inconsistencies in our confirmatory and exploratory outcomes, we built skip patterns into the online questionnaire – if participants indicated they had not had a particular type of sex in the past three months, they were skipped out of more specific questions related to that type of sex. In addition, participants were precluded from indicating they had a particular type of sex without a condom more times than they said they had that type of sex. Because of these internal data checks, there were only limited instances when internal inconsistencies could exist in our data.^[[7]](#footnote-8)^

When reviewing for inconsistencies, we restricted our assessment to variables either used to construct our outcome measures or any variables used to directly edit those variables. As such, the only outcome measure in which potential inconsistencies could exist was the *Current LARC Use* measure. A total of 93 inconsistencies were found in the baseline questionnaire and 62 in the 3-month questionnaire. These were instances in which participants reported using no prescription birth control in the past three months in one question but reported current use of prescription birth control in another question.

In the limited situations where internal inconsistencies were identified, all items leading to the inconsistency were flagged. Our benchmark analytic approach was to include data flagged as inconsistent in analysis, because we do not know for certain which values are true and which are inaccurate. However, we also ran sensitivity analyses that excluded these data from the construction of applicable outcome measures to ensure results were consistent with our benchmark approach (see Table S2 for results).

Identify and Flag Unreliable Cases

The final step in data screening was identifying and flagging entire cases (i.e., entire questionnaires) that were unreliable.^[[8]](#footnote-9)^ By unreliable, we mean that we had sufficient reason to believe that the respondent’s answers were not honest representations of their behaviors, knowledge, and beliefs. In total, we identified one unreliable record at baseline and zero in the three-month follow-up questionnaires. Data for cases that were deemed unreliable were treated as *unit missing* and excluded from benchmark analyses. However, sensitivity analyses that include the unreliable data were conducted (see Table S2 for results).^[[9]](#footnote-10)^

Missing Data Approach

Our six-step decision process for addressing missing data is informed by practical guidance from the Institute of Education Sciences.^[[10]](#footnote-11)^ Our benchmark approach aimed to mitigate the introduction of bias into our impact estimates, provide good estimates of uncertainty, and maximize the use of available data by adjusting missing baseline/covariate data.

1. Implemented the first step in the *Data screening* procedures and reviewed for invalid data. Any data found to be invalid were re-coded to item missing.
2. Determined if logical edits are possible for any analytic variables that may have missing values (due to nonresponse or skip patterns) and logically imputed where possible.
3. Implemented the remaining *Data screening* procedures to identify outlying or inconsistent data in any items used to construct outcome variables and identify unreliable records. Outlying and inconsistent items were flagged, but values remained in our benchmark approach. Unreliable records at a particular time point were treated as unit missing and not included in analysis.
4. Determined if any individuals who were in the randomized sample had no data at all at the three-month follow-up time point. If this was the case, we used case deletion as the literature suggests that this is the most straightforward and prudent approach for missing follow-up data.^[[11]](#footnote-12)^ Attrition statistics are reported.
5. Determined if any individuals who were in the randomized sample (for each confirmatory outcome) were missing baseline covariates or the baseline measure of the outcome variable. If this was the case, our benchmark approach was to use dummy variable adjustment procedures as the literature suggests that this is the most straightforward and prudent approach.^[[12]](#footnote-13)^ Specifically, continuous variables missing a value were imputed to the mean for the sample and dichotomous variables missing a value were imputed to zero. We created missing value dummy variables indicating records where values were missing (before imputation) for a particular variable, coded as 1 if originally missing or 0 when not originally missing. These missing value indicator variables were included in our benchmark analytic approach as covariates in our models.

To explore the potential impact of this approach on our effect estimates, we conducted sensitivity analyses by estimating results with missing baseline data excluded from the analysis (i.e., used case deletion for all cases with missing baseline covariate and missing baseline outcome data). See Table S2 for results.

Data Analysis

Baseline Equivalence Assessment

Baseline equivalence was analyzed for all baseline measures of each outcome variable and prespecified demographic and sexual behavioral measures for each analytic sample. The analytic samples were defined as all participants who were randomized into either the treatment or control conditions and who reported outcome data for a confirmatory measure at the three-month follow-up time point.

We first list and describe the prespecified measures used to examine the equivalence of our treatment and control groups at baseline. After identifying the measures, we provide details on the diagnostic methods that were used to assess any baseline difference that existed between the treatment and control groups in the measures outlined below.

Demographic and Sexual Behavior Measures

Baseline equivalence was assessed for four demographic variables and one baseline measure of sexual behavior (identified below). Each of the variables were constructed from participant self-responses to questions in either the *Eligibility Screening Form* or Video Health Study (VHS) baseline *Participant Questionnaire*. For the race variables, categorical responses to a single question were used to create multiple dichotomous variables. The sexual behavioral variable was constructed from participants’ responses to three questions in the baseline *Participant Questionnaire.* We provide details on variable coding below.

Demographic:

- Age at screening (continuous; range 18–19)^[[13]](#footnote-14)^
- Identify as Black/African American at screening (0 = identify as another race/do not identify race; 1 = identify as Black/African American)^[[14]](#footnote-15)^
- Identify as Hispanic, Latino, or of Spanish origin at screening (0 = do not identify as Hispanic, Latino, or of Spanish origin; 1 = identify as Hispanic, Latino, or of Spanish origin)
- Completed high school (0 = has not completed high school; 1 = has completed high school)

Sexual Behavior:

- Ever had sex (0 = never had vaginal, anal, or oral sex; 1 = has had vaginal, anal, and/or oral sex in lifetime)

Baseline Outcome Measures

In addition to the demographic and sexual behavior measures, we assessed baseline equivalency of the baseline observations of the outcome measures. We provide details on variable coding below.

- *Times having sex without condoms in the past 3 months* at baseline (continuous; values range 0 to *k*, where 0 = has had sex without condoms 0 times in past 3 months and *k* = number of times having sex without condoms in past 3 months)
- *Current LARC use* at baseline (0 = not currently using a LARC; 1 = currently using a LARC)
- *STI Testing in the past 3 months* at baseline (0 = not tested for STIs/STDs in past 3 months; 1 = tested for STIs/STDs in past 3 months)

Balance Assessment Methods

We assessed baseline equivalence of the treatment and control groups according to a multistep procedure. Baseline equivalence statistics were produced for each analytic sample.^[[15]](#footnote-16)^ Only participants who provided a response to all of the items used to construct the confirmatory outcome measure at the three-month follow-up time point were included in the analytic sample for that outcome measure; thus, the analytic samples used for each research question varied slightly because of the exclusion of nonresponders. We report the adjusted means and standardized mean difference of each baseline variable for the treatment and control groups.

To establish baseline equivalence, we generated model-based point estimates of the difference between the treatment and control groups for the identified baseline equivalence variables using the following procedures:^[[16]](#footnote-17)^

- **Step 1*.*** First, we generated a model-based estimate of the difference between treatment and comparison groups on the preintervention measures identified above. Separate models were run for each of the variables. For continuous measures, we used ordinary least squares (OLS) models. For dichotomous measures, we used linear probability models to estimate the predicted probability of group membership. The models are reduced-form variations of the models that we use to estimate program impact.^[[17]](#footnote-18)^

$${Y_{baseline}= \beta}_{0}{+ \beta}_{1}T+\sum\left( \beta_{P}X_{P} \right)+\varepsilon$$

where:

$Y_{baseline}$ – is the baseline measure of the variable that we use to establish baseline equivalency. This variable was included as a covariate in the analytic model. Separate models were estimated for each baseline equivalency measure specified above.

$T$– A dummy treatment indicator variable whose value equaled 1 if the participant was randomized into the treatment group and zero otherwise.

$X$– Region (blocking variable) – An *n* – 1 vector of region indicator dummy variables that were coded 1 if the intervention was delivered at region *n* and coded zero otherwise.

$\beta_{0}$ – The intercept term, which represented the adjusted mean value of the baseline equivalency measure for participants in the control sample, with all other variables in the model held constant at zero.

$\beta_{1}$ – This represented the adjusted (but not standardized) mean difference in the baseline equivalency variable between treatment and control participants.

$\varepsilon$– The residual or random variation that remains for each observation after the structural components of the model are estimated. It is the difference between the observed and the predicted values at the individual level.

- **Step 2.** Reported the adjusted means for the baseline variable of interest for the treatment and control groups.
- **Step 3.** If the baseline measure was continuous, we used the following formula to calculate the pooled within-group standard deviation of the outcome measure:

$$S_{p}=\sqrt{\frac{\left( n_{t}-1 \right)S_{t}^{2}+\left( n_{c}-1 \right)S_{c}^{2}}{(n_{t}+ n_{c}-2)}}$$

where *n_t_* and *n_c_* are the sample sizes, and *S_t_* and *S_c_* are the participant-level standard deviations for the baseline measures for the analytic treatment and comparison groups, respectively. We produced separate calculations of the pooled standardized deviation for each variable used to establish baseline equivalence (as noted above).

- **Step 4.** Produced the standardized difference of means. If the baseline measure was continuous, we used Hedges’ *g* as the formula to compute the standardized difference of means for the treatment and comparison groups:

$$g= \frac{\beta_{1}}{S_{p}}$$

where $\beta_{1}$is the adjusted mean difference in the variable selected to establish baseline equivalence for the treatment and comparison groups (calculated in Step 1), and *S_p_* is the pooled standard deviation (produced in Step 3).

For dichotomous baseline variables we used the Cox Index, which yields effect size values similar to the values of Hedges’ *g* that one would obtain when group means, standard deviations, and sample sizes were available, assuming the dichotomous outcome measure was based on any underlying normal distribution.” Following this guidance, we used the Cox Index to estimate baseline equivalence for dichotomous baseline covariates. The formula is as follows:

$$d_{Cox}= \frac{\left[ ln\left( \frac{p_{t}}{1-p_{t}} \right)-ln\left( \frac{p_{c}}{1-p_{c}} \right) \right]}{1.65}$$

where 𝑝_𝑡_ and 𝑝_𝑐_ represent the probability of occurrence of the event (or characteristic) within the treatment and comparison groups, respectively.

Analytic Approach

We used an intent-to-treat (ITT) framework, which does not measure the effect of the participant’s exposure to the treatment itself but rather the effect of the offer of the treatment relative to the offer of receiving the control condition. This framework maintains the integrity of the experimental structure by including all participants who were randomized (except those who attrite) in the analytic sample, thereby maintaining an exogenous assignment of participants to experimental condition. Bias can be insinuated through self-selection if any participant who was randomized fails to provide outcome data. However, follow-up data collection rates were extremely high (96%) and the differential attrition between treatment and control groups was low (<1%); the literature suggests that a combination of these overall attrition and differential attrition rates result in a tolerable threat of potential bias.^[[18]](#footnote-19)^

Benchmark Model Specification

The confirmatory research questions under investigation in this study were whether offering *Plan A* to participants impacts their (1) reported use of LARC, (2) times having sex without condoms, and (3) reported STI testing. A detailed description of how each of these confirmatory outcome measures were constructed is provided in Table S1. We estimated these impacts using a regression that models intervention effects as a function of assignment to *Plan A* (i.e., treatment), relevant baseline covariates, a baseline measure of the outcome variable, and regional (blocking) indicators. Although a straight difference-of-means approach should provide unbiased estimates of the effect of the treatment, we used a model-based approach because it increases the precision of those estimates.^[[19]](#footnote-20)^ The empirical models were estimated with a negative binomial regression for the one continuous confirmatory outcome measure and logistic regression models for the two dichotomous confirmatory outcome measures. We present the empirical model here:

$${Y_{Post}= \beta}_{0}{+ \beta}_{1}T{+ \beta}_{2}Y_{Pre}+ \sum\left( \beta_{P}X_{P} \right)+ \varepsilon$$

where:

*Y_Post_* – The outcome variable of interest reported by participant *i* at the three-month follow-up (see Table S1 for full details on variable construction).

*Y_Pr_*_e_ – The baseline measure of the outcome variable of interest reported by participant *i* at baseline; variable re-centered at the grand mean for analysis.

*T* – A dummy treatment indicator variable whose value equals 1 if the participant is randomized into the treatment group and 0 otherwise.

*X* – A *p* vector of baseline (i.e., measured prior to receiving intervention) participant-level covariates as well as blocking variables to account for the variation in outcomes associated with these groups. These covariates include (described in detail in the Demographic and Sexual Behaviors Measures section above:

1. Age
2. Black race
3. Hispanic, Latino, or of Spanish ethnicity
4. High school education
5. Region^[[20]](#footnote-21)^

$\beta_{0}$ – The intercept term, which represents, depending on the outcome measure of interest in the analysis, the outcome for the average control participant with all other variables in the model held constant at their mean.

$\beta_{1}$ – This is the parameter estimate of substantive interest. $\beta_{1}$ represents, depending on the confirmatory outcome measure of interest in the analysis, either (1) the adjusted mean difference in treatment and control participants’ self-reported times having sex without condoms in the past three months at the three-month follow-up; (2) the adjusted effect estimate comparing treatment participants’ current LARC use to control participants’ use at the three-month follow-up; or (3) the adjusted effect estimate comparing treatment participants’ STI testing in the past three months to control participants at the three-month follow-up.

$\varepsilon-$The error term or unexplained individual-level variance that remains for each observation after the structural components of the model are estimated. It is the difference between the observed and the predicted values at the individual level.

We report model-estimated effects and the results of significance tests in the paper marginal effects calculated at the mean value for all covariates for ease in interpretation.^[[21]](#footnote-22)^ Statistical significance is based on test statistics produced by Stata for the coefficient $\beta_{1}$ using a two-tailed test, with *p* < .05. We did not adjust for multiple comparisons in our confirmatory outcome measures because each of the three measures falls into its own domain as defined by the *Identifying Programs That Impact Teen Pregnancy, Sexually Transmitted Infections, and Associated Sexual Risk Behaviors, Review Protocol Version 5.0*.^[[22]](#footnote-23)^

Sensitivity Analyses

We conducted sensitivity analyses to test the robustness and validity of our benchmark approach outlined above. The results of sensitivity analyses are reported in Table S2. These include:

1. **Without baseline covariates** **(Model A):** We removed all covariates from the benchmark models, simply regressing the outcomes on the treatment variable.
2. **With expanded baseline covariates (Model B):** In addition to the benchmark covariates, we added the following variables measured at baseline as covariates to the benchmark model: times having sex without condoms in the past three months, current LARC use, STI test in the past three months, HIV test in the past three months, HIV/STI risk perception, pregnancy risk perception, contraceptive knowledge, and ever had sex.
3. **Without imputed baseline data (Model C):** We ran the benchmark models with reduced analytic samples that did not include any imputed baseline data.
4. **Robust OLS (Model D):** Our benchmark approach was to use negative binomial regression for count outcomes and logistic regression for dichotomous outcomes. We assessed the robustness of this decision by also running robust OLS models for all outcome measures (regardless of whether dichotomous or count).
5. **With unreliable data (Model E):** We ran the benchmark models with reduced analytic samples that did not include any observations that were indicated as unreliable (according to the procedures outlined above).
6. **Without late responders (Model F):** Our benchmark approach was to include follow-up data from all participants who completed a questionnaire during their open data collection window, regardless of the time point in that window when it was completed. Data collection windows were open for four months and were intentionally broad to minimize attrition from the analytic sample. To examine whether results were robust to this decision, we conducted sensitivity analyses that excluded late responders from the benchmark analytic sample. Late responders were defined as those participants who completed their three-month questionnaire more than one month after the initiation of the three-month data collection window.
7. **Without outliers (Model G):** We ran the benchmark models with reduced analytic samples that excluded all cases with outcome or baseline outcome values flagged as outliers (according to the procedures outlined above).
8. **Without inconsistent data (Model H):** We ran benchmark models with reduced analytic samples that excluded all cases with outcome or baseline outcome values flagged as inconsistent (according to the procedures outlined above).

***Table S1.*** *Confirmatory and Exploratory Outcome Measures*

| **Outcome Name** | **Description of the Outcome, Including How It Is Operationalized** |
| --- | --- |
| ***CONFIRMATORY OUTCOME MEASURES*** | |
| **Current LARC use** | Data for this measure came from the 3-month follow-up *Participant Questionnaire*. The protective outcome was operationalized as a dichotomous variable indicating whether a person reported currently using LARC (implant or Intra-Uterine Device) or not currently using LARC.  The measure was calculated from the following item:   - Which of the following methods of prescription birth control are you currently using?   - None: I am not currently using any of these methods   - Oral contraceptives (e.g., the pill)   - The patch (e.g., Ortho Evra)   - The shot/injection (e.g., Depo-Provera)   - The ring (e.g., NuvaRing)   - The implant (e.g., Implanon or Nexplanon)   - IUD (e.g., Paragard, Skyla, or Mirena)   A person who selected either *The implant* or *IUD* was given a value of 1 for the measure. A person who selected *None*, *Oral contraceptives, The patch*, *The shot/injection*, or *The ring* was given a value of 0 for the measure. The resulting variable was dichotomous with values 0 or 1, where 0 indicates a person who does not currently use a LARC and 1 indicates a person who does currently use a LARC. |
| **Times having sex without condoms** | Data for this measure came from the 3-month follow-up *Participant Questionnaire.* The risk outcome was operationalized as the number of times in the past 3 months a person reports having any type of sex without using a condom.  The measure was calculated from the following items:   - In the past 3 months, how many times have you had vaginal sex without using a condom? - In the past 3 months, how many times have you had oral sex without using a condom? - In the past 3 months, how many times have you had anal sex without using a condom?   The measure was calculated by summing the total number of times a person reported not using a condom during vaginal, oral, and anal sex in the past 3 months. The resulting variable was continuous with values that range from 0 to *k*, where 0 indicates that a person has not engaged in sex without a condom in the past 3 months, and *k* indicates the number of times the person has engaged in sex without a condom (risk behavior) in the past 3 months. |
| **STI testing** | Data for this measure came from the 3-month follow-up *Participant Questionnaire*. The protective outcome was operationalized as a dichotomous variable indicating whether a person reported having been tested for STIs/STDs in the past 3 months or not having been tested.  The measure was calculated from the following item:   - Have you been tested for STIs/STDs other than HIV in the past 3 months?   A person who selected either *Yes* was given a value of 1 for the measure. A person who selected *No* was given a value of 0 for the measure. The resulting variable was dichotomous with values 0 or 1, where 0 indicates a person who has not been tested for STIs/STDS in the past 3 months and 1 indicates a person who has been tested for STIs/STDs. |
| ***EXPLORATORY MEASURES*** | |
| **HIV testing** | Data for this measure came from the 3-month follow-up *Participant Questionnaire*. The protective outcome was operationalized as a dichotomous variable indicating whether a person reported having been tested for HIV in the past 3 months or not having been tested.  The measure was calculated from the following item:   - Have you been tested for HIV in the past 3 months?   A person who selected *Yes* was given a value of 1 for the measure. A person who selected *No* was given a value of 0 for the measure. The resulting variable was dichotomous with values 0 or 1, where 0 indicates a person who has not been tested for HIV in the past 3 months and 1 indicates a person who has been tested for HIV. |
| **Contraceptive knowledge** | Data for this measure came from the 3-month follow-up *Participant Questionnaire*. The outcome was operationalized as a proportionate variable indicating the percentage of items to which a person provided an accurate response in a series of *True/False* questions out of all total questions.  The measure was calculated from the following items:   - Birth control pills are effective even if a woman misses taking them for 2 or 3 days in a row. (Correct response: *False*) - For people who are sexually active, if used properly, condoms are the only type of birth control that will protect you from unplanned pregnancy and sexually transmitted infections (STIs). (Correct response: *True)* - In order to get birth control, a woman must have a pelvic exam. (Correct response: *False*) - Women using the birth control shot (e.g., Depo-Provera) must get an injection from a health care provider every 3 months. (Correct response: *True*) - Women using the vaginal ring (e.g., NuvaRing) must have it inserted by a doctor or health care provider every month. (Correct response: *False*) - Long-acting methods of birth control like the implant or IUD cannot be removed early, even if a woman changes her mind about wanting to get pregnant. (Correct response: *False*) - Women using the birth control patch (e.g., Ortho Evra) must apply a new patch each day. (Correct response: *False*) - Emergency contraception (sometimes called the morning after pill or Plan B must be taken within 24 hours after having unprotected sex to be effective. (Correct response: *False*) - To obtain an IUD, a woman must undergo a surgical operation. (Correct response: *False*) - Condoms have an expiration date. (Correct response: *True*)   A person who selected the correct response for an item was given a value of 1 for that item. A person who selected the incorrect response for an item or left the item missing was given a value of 0 for that item. The correct items were then summed and divided by the total number of items in the scale. The resulting variable was continuous with values ranging from 0 to 1, indicating the percentage of correct items selected. |
| **HIV/STI risk perception** | Data for this measure came from the 3-month follow-up *Participant Questionnaire*. The outcome measure was operationalized as a mean scale score indicating the person’s perceived likelihood of their getting HIV and STIs in the next year.  The measure was calculated from the following items:   - If, in the next year, you were to have sex (vaginal, oral, or anal) for a month without using a condom, how likely is it that you would get HIV? - If, in the next year, you were to have sex (vaginal, oral, or anal) for a month without using a condom, how likely is it that you would get an STI (other than HIV)?   Responses to each item in the scale were assessed using a 7-point semantic differential scale ranging from 1 = *very unlikely* to 7 = *very likely*. Individuals’ responses to the two items were summed and averaged to generate a mean scale score. The resulting variable was continuous with values ranging from 1 to 7, with a score of 1 indicating the lowest perceived likelihood of getting HIV/STIs and a score of 7 indicating the highest perceived likelihood of getting HIV/STIs in the next year. |
| **Pregnancy risk perception** | Data for this measure came from the 3-month follow-up *Participant Questionnaire*. The outcome measure was operationalized as a mean scale score indicating the person’s perceived likelihood of their getting pregnant in the next year.  The measure was calculated from the following items:   - If, in the next year, you were to have vaginal sex with someone just once without using any birth control, how likely is it that you would get pregnant? - If, in the next year, you were to have vaginal sex for a month without using any form of birth control, how likely is it that you would get pregnant?   Responses to each item in the scale were assessed using a 7-point semantic differential scale ranging from 1 = *very unlikely* to 7 = *very likely*. Individuals’ responses to the two items were summed and averaged to generate a mean scale score. The resulting variable was continuous with values ranging from 1 to 7, with a score of 1 indicating the lowest perceived likelihood of getting pregnant and a score of 7 indicating the highest perceived likelihood of getting pregnant in the next year. |
| **Discussion of sexual/reproductive health topics** | Data for these measures came from the *Post-visit Questionnaire*. The outcomes were operationalized as dichotomous variables indicating whether a person reported talking with their health care provider about each topic or not during the baseline sexual/reproductive health visit.  The measures were calculated from the following select-all-that-apply item:   - We would like to know about what you and your health care provider(s) talked about during today’s visit. Below we list a number of topics related to your sexual/reproductive health; please indicate which of these topics you talked about or learned about during today’s visit.   - IUDs (e.g., Paragard, Skyla, or Mirena)   - Birth control implants (e.g., Implanon or Nexplanon)   - Condoms   - Other birth control methods (e.g., the pill, the path, or the ring)   - Dual methods of protection (using condoms and another form of birth control at the same time)   - Getting tested for HIV or other sexually transmitted infections/diseases (STIs/STDs), such as chlamydia or gonorrhea   - Risks associated with sexual behaviors (e.g., unplanned pregnancy, HIV, or STIs)   A person who selected a topic was given a value of 1 for the measure. A person who did not select a topic was given a value of 0 for the measure. The resulting variable was dichotomous with values 0 or 1, where 0 indicates a person who did not discuss the topic at their baseline sexual/reproductive health visit and 1 indicates a person who did discuss the topic. |
| **Discussion of your sexual behaviors** | Data for this measure came from the *Post-visit Questionnaire*. The outcome was operationalized as dichotomous variables indicating whether a person reported talking with their health care provider about their sexual behaviors or not during the baseline sexual/reproductive health visit.  The measure was calculated from the following item:   - Did you and your health care provider(s) discuss your sexual behaviors (things you have personally done, like having sex or using condoms) during today’s visit?   A person who selected *Yes* was given a value of 1 for the measure. A person who selected *No* was given a value of 0 for the measure. The resulting variable was dichotomous with values 0 or 1, where 0 indicates a person who did not discuss their sexual behaviors at their baseline sexual/reproductive health visit and 1 indicates a person who did discuss their sexual behaviors. |
| **Ask questions or mention concerns** | Data for this measure came from the *Post-visit Questionnaire*. The outcome was operationalized as dichotomous variables indicating whether a person reported asking questions or mentioning concerns during the baseline sexual/reproductive health visit.  The measure was calculated from the following item:   - Did you ask any questions or mention any concerns you have to your health care provider(s) during today’s visit?   A person who selected *Yes* was given a value of 1 for the measure. A person who selected *No* was given a value of 0 for the measure. The resulting variable was dichotomous with values 0 or 1, where 0 indicates a person who did not ask questions or mention concerns at their baseline sexual/reproductive health visit and 1 indicates a person who did ask questions or mention concerns. |
| **Comfort level talking with provider about your sexual health** | Data for this measure came from the *Post-visit Questionnaire*. The outcome measure was operationalized as a score indicating the person’s reported comfort level talking with their health care provider about their sexual/reproductive health during the baseline sexual/reproductive health visit.  The measure was calculated from the following item:   - On a scale from 1 to 5, how comfortable did you feel talking with your health care provider(s) about your sexual/reproductive health during today’s visit?   Responses to the item were assessed using a 5-point semantic differential scale ranging from 1 = *Non at all comfortable* to 5 = *extremely comfortable*. The resulting variable was continuous with values ranging from 1 to 5, with a score of 1 indicating the lowest level of comfort and a score of 5 indicating the highest level of comfort. |
| **Comfort level talking with provider about your sexual behaviors** | Data for this measure came from the *Post-visit Questionnaire.* The outcome measure was operationalized as a score indicating the person’s reported comfort level talking with their health care provider about their sexual behaviors during the baseline sexual/reproductive health visit.  The measure was calculated from the following item:   - On a scale from 1 to 5, how comfortable did you feel talking with your health care provider(s) about your sexual behaviors during today’s visit?   Responses to the item were assessed using a 5-point semantic differential scale ranging from 1 = *not at all comfortable* to 5 = *extremely comfortable*. The resulting variable was continuous with values ranging from 1 to 5, with a score of 1 indicating the lowest level of comfort and a score of 5 indicating the highest level of comfort. |
| **Satisfaction level with provider** | Data for this measure came from the *Post-visit Questionnaire*. The outcome measure was operationalized as a score indicating the person’s reported satisfaction level with their health care provider during the baseline sexual/reproductive health visit.  The measure was calculated from the following item:   - On a scale from 1 to 5, at the end of today’s visit, how satisfied were you with the way your provider(s) addressed your questions or concerns?   Responses to the item were assessed using a 5-point semantic differential scale ranging from 1 = *not at all satisfied* to 5 = *extremely satisfied*. The resulting variable was continuous with values ranging from 1 to 5, with a score of 1 indicating the lowest level of satisfaction and a score of 5 indicating the highest level of satisfaction. |

***Table S2.*** *Sensitivity Analyses for Confirmatory and Exploratory Three-Month Follow-up Outcome Measures*

|  | **Benchmark Model** | | **Model A: Without Baseline Covariates** | | **Model B: With Expanded Baseline Covariates** | | **Model C: Without Imputed Baseline Data** | | **Model D: Robust OLS** | | **Model E: With Unreliable Data** | | **Model F: Without Late Responders** | | **Model G: Without Outliers** | | **Model H: Without Inconsistent Data** | |
| --- | --- | --- | --- | --- | --- | --- | --- | --- | --- | --- | --- | --- | --- | --- | --- | --- | --- | --- |
| **​Variable** | **​Effect Estimate (Standard Error)^a^** | **​*p*** | **​ Effect Estimate (Standard Error)^a^** | ***p*** | **​Effect Estimate (Standard Error)^a^** | **​*P*** | **​Effect Estimate (Standard Error)^a^** | **​*p*** | **​Effect Estimate (Standard Error)^a^** | **​*p*** | **​Effect Estimate (Standard Error)^a^** | **​*p*** | **​Effect Estimate (Standard Error)^a^** | **​*p*** | **​Effect Estimate (Standard Error)^a^** | **​*p*** | **​Effect Estimate (Standard Error)^a^** | **​*p*** |
| **Behavioral Outcomes** | | |  |  |  |  |  |  |  |  |  |  |  |  |  |  |  |  |
| **​Current LARC use^a^** | 0.17 (0.145) | 0.230 | 0.08 (0.128) | 0.512 | 0.18 (0.145) | 0.227 | 0.18 (0.145) | 0.204 | 0.02 (0.016) | 0.251 | 0.17 (0.145) | 0.230 | 0.15 (0.155) | 0.348 |  |  | 0.23 (.148) | 0.115 |
| **Times having sex without condoms in past 3 months^a^** | –0.04 (0.063) | 0.504 | –0.08 (0.076) | 0.296 | –0.06 (0.062) | 0.314 | –0.05 (0.064) | 0.407 | –0.66 (1.235) | 0.591 | –0.04 (0.063) | 0.503 | –0.04 (0.067) | 0.587 | –0.02 (0.056) | 0.750 |  |  |
| **STI test in past 3 months^a^** | 0.19 (0.099) | 0.053 | **0.20 (0.097)** | **0.038** | **0.20 (0.101)** | **0.045** | 0.19 (0.100) | 0.063 | 0.05 (0.024) | 0.054 | **0.20 (0.099)** | **0.049** | 0.20 (0.107) | 0.058 |  |  |  |  |
| **HIV Test in past 3 months** | 0.19 (0.105) | 0.073 | 0.18 (0.101) | 0.077 | 0.19 (0.106) | 0.073 | 0.18 (0.105) | 0.081 | 0.04 (0.023) | 0.073 | 0.19 (0.105) | 0.070 | 0.15 (0.111) | 0.170 |  |  |  |  |
| **Current LARC use (first-time SRH users only)** | **0.53 (0.259)** | **0.040** | **0.56 (0.256)** | **0.029** | **0.57 (0.265)** | **0.032** | **0.54 (0.259)** | **0.038** | **0.07 (0.034)** | **0.038** | **0.56 (0.256)** | **0.029** | **0.52 (0.268)** | **0.053** |  |  | **0.68 (0.266)** | **0.010** |
| **Behavioral Antecedents** | | |  |  |  |  |  |  |  |  |  |  |  |  |  |  |  |  |
| **Contraceptive knowledge** | **0.03 (0.007)** | **<0.001** | **0.04 (0.009)** | **<0.001** | **0.03 (0.007)** | **<0.001** | **0.03 (0.007)** | **<0.001** | **0.03 (0.007)** | **<0.001** | **0.03 (0.007)** | **<0.001** | **0.03 (0.008)** | **<0.001** |  |  |  |  |
| **HIV/STI risk perception** | 0.18 (0.093) | 0.057 | **0.25 (0.110)** | **0.026** | 0.18 (0.093) | 0.058 | 0.16 (0.093) | 0.087 | 0.18 (0.093) | 0.057 | 0.18 (0.093) | 0.057 | 0.17 (0.099) | 0.090 |  |  |  |  |
| **Pregnancy risk perception** | 0.07 (0.079) | 0.389 | 0.10 (0.086) | 0.227 | 0.06 (0.078) | 0.431 | 0.05 (0.079) | 0.492 | 0.07 (0.079) | 0.390 | 0.07 (0.079) | 0.394 | 0.06 (0.082) | 0.454 |  |  |  |  |

^a^ Denotes confirmatory outcomes.

1. The one exception to the benchmark analytic approach, when applied to exploratory outcome measures, was in the regression model used for the continuous exploratory measures. When assessing the potential impact of *Plan A* on these exploratory continuous outcome measures, we used ordinary least squares (OLS) regression. [↑](#footnote-ref-2)
2. Prior to conducting analysis of the data, the *Analysis Plan* indicated that identification of invalid responses would be the second step in our data screening process. However, once analysis began, it became clear that it was first important to ensure that values for all items in the questionnaire were consistent with the codebook, or corrected to the accurate value, before proceeding with the remaining data screening steps. [↑](#footnote-ref-3)
3. Regardless of whether or not data were nominal, ordinal, or continuous, all response options were coded in Stata as numeric values; values were labeled according to corresponding category names when data were nominal or ordinal. As an example, the variable gender is a nominal variable; however, it is treated as a dummy variable where females are coded as 1 and males are coded as 0. The only acceptable values for this variable then are 0 and 1; any other values are out of range. [↑](#footnote-ref-4)
4. Rules for identifying outliers are informed by the following: Hamilton, L. C. 2006. *Statistics with STATA: Updated for version 9 and NIST/SEMATECH e-handbook of statistical methods*, http://www.itl.nist.gov/div898/handbook/ [↑](#footnote-ref-5)
5. Prior to conducting analysis of the data, the *Analysis Plan* stated that we would also review for over-time inconsistencies. Over-time inconsistencies refer to instances in which lifetime reported behaviors decline or are completely recanted over time. For example, a respondent who at baseline indicated that she has had vaginal sex in her life, but on a subsequent administration of the questionnaire said that she has never had vaginal sex. However, after internal study team discussion, it was decided that because construction of the confirmatory outcome measures uses data from only one time point (i.e., three-month follow-up questionnaire), the only data that should be informing the construction of those variables should be data reported in the three-month follow-up questionnaire. [↑](#footnote-ref-6)
6. Inconsistencies can occur for a number of reasons including social desirability bias and memory or recall issues on the part of the respondent and misunderstanding on the part of either the respondent or interviewer. These issues are especially common in self-reports of sexual behaviors where questions are of a sensitive nature, and often respondents are asked to indicate the frequency and/or recency of behaviors over differing lengths of time (e.g., 30 days, 3 months, 6 months). See the following sources for more information on inconsistencies in reporting: Alexander, C. S., Somerfield, M. R., Ensminger, M. E., Johnson, K. E., & Kim, Y. J. (1993). Consistency of adolescents’ self-report of sexual behavior in a longitudinal study. *Journal of Youth and Adolescence*, *22*(5), 455–471. Clarke, P. M., Fiebig, D. G., & Gerdtham, U. G. (2008). Optimal recall length in survey design. *Journal of Health Economics*, *27*(5), 1275–1284. Del Boca, F. K., & Noll, J. A. (2000). Truth or consequences: The validity of self-report data in health services research on addictions. *Addiction*, *95*(11), S347–S360. Harris, K. M., Griffin, B. A., McCaffrey, D. F., & Morral, A. R. (2008). Inconsistencies in self-reported drug use by adolescents in substance abuse treatment: Implications for outcome and performance measurements*. Journal of Substance Abuse Treatment*, *34*(3), 347–355. Schroder, K. E., Carey, M. P., & Vanable, P. A. (2003). Methodological challenges in research on sexual risk behavior: II. Accuracy of self-reports. *Annals of Behavioral Medicine*, *26*(2), 104–123. Schwarz, N., & Oyserman, D. (2001). Asking questions about behavior: Cognition, communication and questionnaire construction. *American Journal of Evaluation*, *22*(2), 127–160. [↑](#footnote-ref-7)
7. In rare instances, paper-based questionnaires were completed by participants. This occurred when the study coordinator or research assistant was unable to open the online questionnaire because of network connection issues, or when the participant could not be reached for an online administration and were instead mailed a paper questionnaire. Paper-based questionnaires included the same skip patterns as online questionnaires; however, unlike the online questionnaire where participants are skipped out of subsequent questions based on their response to previous questions and cannot report inconsistent times having different types of sex, it was possible for participants to provide responses to questions that they should not have answered or that did not make sense given their responses to previous questions. [↑](#footnote-ref-8)
8. Prior to conducting analysis of the data, the *Analysis Plan* stated that identification of unreliable cases would be the first step in the data screening process. However, after internal study team discussion, it was determined that this should be the final step in the process, after all other screening procedures had been completed. [↑](#footnote-ref-9)
9. Prior to conducting analysis of the data, the *Analysis Plan* stated that we would review response patterns to in each questionnaire to determine whether there were any perceptible clear, deliberate patterns. However, after internal team discussion, it was decided that because questionnaire completion was largely done online, where it would be challenging for participants to purposively use a patterned response, this was an unnecessary step in determining reliability. [↑](#footnote-ref-10)
10. Puma, M. J., Olsen, R. B., Bell, S. H., & Price, C. (2009). *What to do when data are missing in group randomized controlled trials*. Washington, DC: National Center for Education Evaluation and Regional Assistance, Institute of Education Sciences, U.S. Department of Education. [↑](#footnote-ref-11)
11. See footnote 10. [↑](#footnote-ref-12)
12. See footnote 10. [↑](#footnote-ref-13)
13. Age at screening was determined using the participant’s self-reported date of birth. [↑](#footnote-ref-14)
14. At screening, participants were asked, “What is your race and ethnicity?” and provided with a list of the following response options: *White*; *Black or African American*; *Hispanic, Latino, or Spanish origin*; *American Indian or Alaskan Native*; *Asian*; *Native Hawaiian/Other Pacific Islander*; *Unknown*; or *Some other race/ethnicity*. Participants could select more than one category and they could also specify some other race/ethnicity. To be eligible for the study, participants had to select either *Black/African American* or *Hispanic, Latino, or Spanish origin*. [↑](#footnote-ref-15)
15. Due to item missing outcome data, there are slight differences in analytic samples for each research question. [↑](#footnote-ref-16)
16. Note that for our benchmark approach, we produced diagnostic estimates of baseline equivalence on the exact same analytic samples that were used in our confirmatory analyses. We assessed equivalence using nonimputed baseline data, and then ran a sensitivity analysis where we assessed equivalency for the same samples using imputed baseline data. [↑](#footnote-ref-17)
17. It is a reduced form because individual-level, demographic covariates are omitted. It is a variation because the dependent variable is the baseline equivalence variable, not the outcome measure. [↑](#footnote-ref-18)
18. What Works Clearinghouse. (n.d.). *Standards handbook version 4.0.* Available at: https://ies.ed.gov/ncee/wwc/Docs/referenceresources/wwc_standards_handbook_v4.pdf. Accessed January 5, 2021. [↑](#footnote-ref-19)
19. We chose to use regression-based models that include covariates, because randomization should ensure covariates are uncorrelated with the treatment variable (i.e., they should not affect the estimate of the treatment effect), but in the instance they are significant predictors of the outcome, their inclusion in a regression model will decrease the standard error of the estimates, making them more precise. See Angrist, J. D., & Pischke, J.-S. (2009). *Mostly harmless econometrics: An empiricist’s companion*. Princeton, NJ: Princeton University Press. Rosenblum, M., & van der Laan, M. J. (2009). Using regression models to analyze randomized trials: Asymptotically valid hypothesis tests despite incorrectly specified models. *Biometrics*, *65*, 937–945. doi:10.1111/j.1541-0420.2008.01177.x. [↑](#footnote-ref-20)
20. Health centers that participated in the study were organized into four regions for administrative and staffing purposes. [↑](#footnote-ref-21)
21. Marginal effects are calculated at the mean of all variables in the model. The marginal effect represents the conditional predicted change in probability (for binary outcomes) and change in count (for count outcomes) associated with being assigned to the treatment group (versus the control group). [↑](#footnote-ref-22)
22. In our registered *Analysis Plan* on clinicaltrials.gov, we indicated we would correct for multiple comparisons in our confirmatory outcomes regardless of domain because we understood multiple comparison correction to be an expectation of the grant. However, further guidance was provided after the *Analysis Plan* was submitted, which indicated that multiple comparison corrections were not expected if outcomes were in separate domains. Mathematica Policy Research. (April 2016). *Identifying Programs That Impact Teen Pregnancy, Sexually Transmitted Infections, and Associated Sexual Risk Behaviors, Review Protocol Version 5.0*. Available at: https://tppevidencereview.youth.gov/pdfs/tpper_review%20protocol_v5.pdf. Accessed January 5, 2021. [↑](#footnote-ref-23)
